# Current Perspectives on Kisspeptins Role in Behaviour

**DOI:** 10.3389/fendo.2022.928143

**Published:** 2022-06-09

**Authors:** Edouard G. Mills, Lisa Yang, Ali Abbara, Waljit S. Dhillo, Alexander N. Comninos

**Affiliations:** ^1^ Section of Endocrinology and Investigative Medicine, Imperial College London, London, United Kingdom; ^2^ Department of Endocrinology, Imperial College Healthcare National Health Service (NHS) Trust, London, United Kingdom

**Keywords:** kisspeptin, KISS1, behaviour, sex, mood, emotions

## Abstract

The neuropeptide kisspeptin is now well-established as the master regulator of the mammalian reproductive axis. Beyond the hypothalamus, kisspeptin and its cognate receptor are also extensively distributed in extra-hypothalamic brain regions. An expanding pool of animal and human data demonstrates that kisspeptin sits within an extensive neuroanatomical and functional framework through which it can integrate a range of internal and external cues with appropriate neuroendocrine and behavioural responses. In keeping with this, recent studies reveal wide-reaching effects of kisspeptin on key behaviours such as olfactory-mediated partner preference, sexual motivation, copulatory behaviour, bonding, mood, and emotions. In this review, we provide a comprehensive update on the current animal and human literature highlighting the far-reaching behaviour and mood-altering roles of kisspeptin. A comprehensive understanding of this important area in kisspeptin biology is key to the escalating development of kisspeptin-based therapies for common reproductive and related psychological and psychosexual disorders.

## Introduction

From an evolutionary perspective, the reproductive system is the most fundamental biological system obligate for species survival. In view of this, the behavioural strategies to facilitate reproduction must be highly efficient and meticulously coordinated (1). In many species, the timing of reproductive activity is coordinated by the integration of internal physiological events and behavioural factors (2). Indeed, in most sexually reproducing species, reproductive behaviour occurs when fertilisation is most likely to happen and is otherwise suppressed (3). Moreover, several species like humans have evolved to gain reward and satisfaction from sex and its precursors (sexual desire and arousal) (4). Taken together, it is therefore conceivable that reproductive behaviour, mood and emotions are mediated by similar hormonal mechanisms, in a presumed drive towards successful reproduction. Although oestrogen and testosterone impact reproductive behaviour circuits (5), emerging data supports the key roles for upstream factors, particularly kisspeptin which is the focus of this review.

The reproductive neuropeptide kisspeptin is a well-established orchestrator of reproductive hormone secretion, acting at the apex of the hypothalamic-pituitary-gonadal (HPG) reproductive axis (6, 7). Emerging data from animal models and human studies reveals that outside of the classical reproductive axis, kisspeptin also modulates reproductive behaviour, mood and emotions, in part at least through its extensive distribution in the limbic system (8–11). In this review, we provide a comprehensive update on the current animal and human literature highlighting the far-reaching behaviour and mood-altering effects of kisspeptin. A comprehensive understanding of this important area in kisspeptin biology is key to the escalating development of kisspeptin-based therapies for common reproductive and related psychological and psychosexual disorders. In addition, we discuss the most recent data examining kisspeptin’s effects on copulatory behaviour and mood in rodents, as well as a broad range of contemporary human evidence, thereby providing an update to the field. Furthermore, we separate out the evidence into pre-clinical animal studies and human studies to permit fuller methodological and cross-species appraisal of the data.

## Methods

We performed a literature review and identified relevant publications by means of a series of PubMed searches for English-language articles. The search terms were (“kisspeptin” OR “KISS1” OR “KISS1R” OR “GPR54”) AND (“behaviour” OR “sex” OR “olfaction/olfactory” OR “audition/auditory” OR “partner/mate preference” OR “lordosis” OR “erection” OR “mood” OR “emotion” OR “depression” OR “anxiety” OR “fear). Relevant data were subsequently extracted from the identified publications, and secondary data sources identified therein. To ensure inclusion of the most up-to-date data, searches were performed up until 25^th^ April 2022.

## Kisspeptin: An Overview

Encoded by the *KISS1* gene in humans (*Kiss1* in non-humans), the kisspeptins are a family of structurally related peptides derived from a common 145-amino acid precursor (10, 11). Although debate exists as to what extent each peptide is naturally present, cleavage gives rise to four circulating fragments: kisspeptin-54, -14, -13 and -10, with the suffix conveying the amino acid length. These four kisspeptin isoforms share a common C-terminal decapeptide sequence (equivalent to kisspeptin-10), conferring the minimum amino acid sequence required for biological activity at a seven transmembrane G protein-coupled receptor, the kisspeptin receptor (encoded by *KISS1R* humans/*Kiss1r* non-humans) (8, 10, 11).

Following its initial discovery as a human malignant melanoma metastasis-suppressor gene by Danny Welch and colleagues in Hershey Pennsylvania (from where its name originates based on the local chocolate sweets) (12), evidence emerged identifying kisspeptin-signalling as essential for reproductive health. Humans with inactivating mutations of *KISS1* or *KISS1R* display a phenotype of hypogonadotropic hypogonadism (6, 7), whereas activating mutations of *KISS1* or *KISS1R* causes central precocious puberty (13). Subsequent physiological studies have been key in defining kisspeptin’s role within the reproductive axis: kisspeptin sits at the apex of the HPG axis stimulating endogenous gonadotropin-releasing hormone (GnRH) release, thereby resulting in release of gonadotropins [luteinizing hormone (LH) and follicle stimulating hormone (FSH)] from the anterior pituitary and subsequent sex steroid release from the gonads (14).

Beyond mammalian species, the kisspeptin system is also found in non-mammalian vertebrates. Indeed, in 2004 the ortholog of the kisspeptin receptor (*Kiss2r*) was first isolated in a teleost species (Nile tilapia), providing the initial evidence of a kisspeptin system in fish (15). Unlike rodents and humans, more than one form of kisspeptin and kisspeptin receptor paralogs have been identified in many fish species. For instance, zebrafish possess two kisspeptin genes (*Kiss1* and *Kiss2*) and two corresponding cognate receptor genes (*Kiss1r* and *Kiss2r*) (16). While both *Kiss1* and *Kiss1r* are co-expressed in the ventral habenula, *Kiss2* expressing neurons reside in the hypothalamic preoptic area and are implicated in gonadotropin secretion (17, 18). However, it is worth noting that several gene mutation studies in zebrafish and medaka reveal that the reproductive role of kisspeptin may be dispensable (19–22), with further studies warranted to examine the possible redundancy of kisspeptin-signalling in reproduction in different species of fish [reviewed extensively and recently in (23)].

## Patterns of Kisspeptin-Signalling

A series of distribution studies have been instrumental in improving our knowledge of central and peripheral kisspeptin expression, thereby providing an anatomical framework for the pleotropic roles of kisspeptin-signalling.

### Central Kisspeptin Expression

#### Hypothalamic Expression

Kisspeptin is expressed in two anatomically discrete hypothalamic nuclei in rodents: the arcuate nucleus (ARC) and the anteroventral periventricular nucleus (AVPV) (24–26). Of note, most kisspeptin neurons in the ARC co-express neurokinin B and dynorphin, which are termed the kisspeptin/neurokinin B/dynorphin (KNDy) neurons (27). The two equivalent regions of kisspeptin expression in the human hypothalamus have been mapped to the infundibular nucleus (corresponding to the rodent ARC) and the rostral preoptic area, with both neuronal populations projecting to GnRH neurons in the mediobasal hypothalamus (28, 29). Interestingly, the limited degree to which these human populations mirror the rodent populations in terms of neurokinin B and dynorphin co-expression suggests species variation (30–32).

#### Extra-Hypothalamic Expression

Kisspeptin-immunoreactive fibres have been identified in the rodent medial amygdala (MeA), bed nucleus of the stria terminalis, paraventricular thalamus and periaqueductal grey (26), as well as *Kiss1r* mRNA detected in the amygdala, frontal cortex, hippocampus, midbrain, pons, striatum and thalamus (8). Moreover, the highest density of kisspeptin receptor expression in the mouse central nervous system can be found in the dentate gyrus of the hippocampus (33). Turning to humans, *KISS1* and *KISS1R* mRNA are widely detected outside the hypothalamus, including the amygdala, caudate nucleus, cingulate gyrus, globus pallidus, hippocampus, medial frontal gyrus, nucleus accumbens, parahippocampal gyrus, putamen, striatum, substantia nigra, superior frontal gyrus and thalamus, as determined by reverse transcription polymerase chain reaction (9, 10). From a functional perspective, these brain structures constitute key limbic and paralimbic regions, which have important functions in controlling mood and behaviour (34) and alludes to kisspeptin’s involvement in these important domains.

### Peripheral Kisspeptin Expression

In rodents, *Kiss1* and *Kiss1r* mRNA has been identified in peripheral regions, including the liver, intestine (particularly the caecum) and bone (8, 35, 36). Moreover, abundant *Kiss1* and *Kiss1r* expression is detected in peripheral reproductive organs in rodents, including ovary (35), uterus (37) and testes (38). Regarding humans, *KISS1* mRNA expression is principally found in the placenta and testis, as well as moderate expression levels in the liver, pancreas, small intestine (9, 11). Moreover, notable *KISS1R* mRNA expression is present in the placenta and pancreas, with lower expression levels in organs such as adipose tissue, kidney, liver, small intestine, spleen, stomach, testis and bone (9–11, 39).

## Reproductive Behaviour

Reproductive behaviour constitutes a complex chain of behavioural adaptations and strategies related to the ultimate production of offspring. It includes the identification of suitable mating partners, courtship, and sexual behaviour, as well as bonding (2). A wealth of literature implicates kisspeptin-signalling in all aspects of reproductive behaviour from rodents through to humans.

### Olfaction

Olfaction underpins many important mating behaviours in mammals through the detection of chemosignals, also known as pheromones. The accessory olfactory bulb is the primary centre for detecting and processing information from chemosignals in animals (40).

#### Preclinical Animal Studies

Using immunofluorescence histochemistry in adult male rats, kisspeptin neurons have been visualised in the mitral cell layer of the accessory olfactory bulb (41). Furthermore, these kisspeptin fibres connect bi-directionally with kisspeptin neuronal fibres in the MeA which in turn, make connections with the preoptic area of the hypothalamus (41). The existence of this neuro-anatomical framework suggests that kisspeptin is a potential integrator of olfactory pheromonal cues with the HPG axis.

To provide functional relevance for the neuroanatomical connections between the olfactory system and kisspeptin populations, animal models have examined the influence of opposite-sex olfactory stimuli on kisspeptin neuronal activity. In ovariectomised female rats, increased kisspeptin activity is observed in the AVPV in response to male (but not female) odours, with associated augmentation of the LH surge (42). Similarly, female mice show activation in AVPV kisspeptin neurons on exposure to male (but not female) urinary pheromones (43). Sexual dimorphism is demonstrated with male mice exhibiting no changes in AVPV or arcuate kisspeptin activity when exposed to female odours (44). Instead, a two-fold rise in the number of c-Fos positive MeA kisspeptin neurons is observed with a concomitant rise in LH release (44).

Olfactory signals are also vital in sheep and goats, whereby the introduction of a stimulus male can override the normal suppressive effects of oestradiol in anoestrous females. Termed the “ram effect”, this sociosexual stimulus is known to be mediated by male pheromones detected by the female (45). In ovariectomised goats, exposure to a sexually mature male is associated with a significant rise in arcuate kisspeptin neuronal activity with concurrent LH pulse generation (46). Similarly, when anoestrous ewes are exposed to a novel male, increased c-Fos activity is seen in arcuate kisspeptin neurons with associated increases in LH amplitude and pulse frequency, an effect which is blocked by pre-administration of a kisspeptin antagonist (47).

#### Human Studies

Using functional neuroimaging [functional Magnetic Resonance Imaging (fMRI)], a recent study demonstrated that peripheral kisspeptin administration enhances limbic brain activity in healthy heterosexual men when exposed to an established feminine scent (Chanel No5) (48). In response to this feminine olfactory stimulus, brain activation was seen in key limbic regions involved in olfactory processing, hedonic valuation of olfactory stimuli and sexual arousal (49). Notably in this study, kisspeptin did not enhance brain activity in control motor areas, indicating specificity of kisspeptin’s effects in olfactory and limbic circuits governing sexual behaviour on exposure to a feminine olfactory stimulus (48).

**Figure 1:**
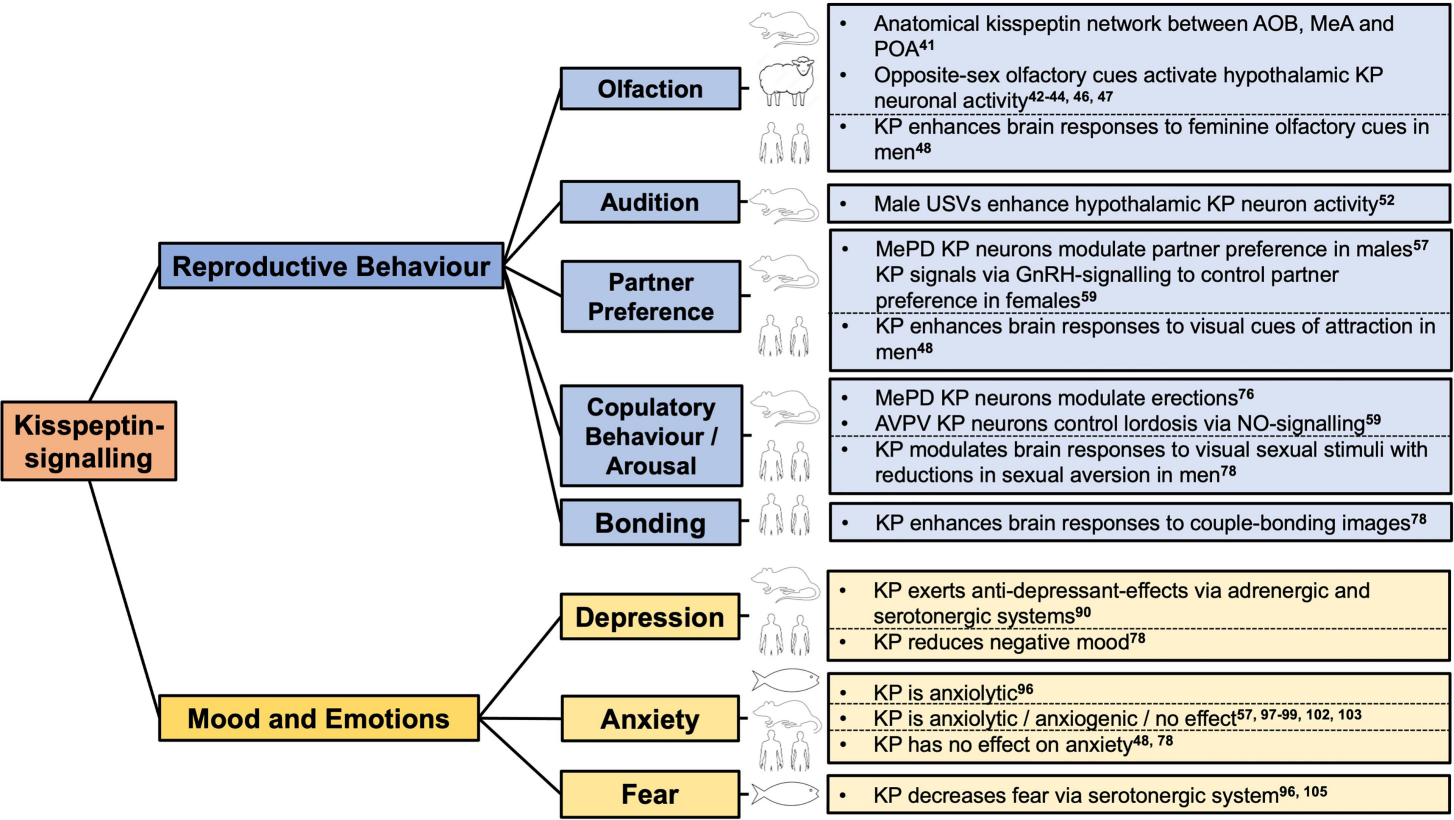
Kisspeptin-signalling has neuromodulatory effects on reproductive behaviour, mood and emotions in zebrafish to humans. AOB, accessory olfactory bulb; AVPV, anteroventral periventricular nucleus; GnRH, gonadotropin releasing hormone; KP, kisspeptin; MeA, medial amygdala; MePD, posterodorsal subnucleus of the medial amygdala; POA, preoptic area; USVs, ultrasonic vocalisations.

Taken together, these findings provide insight into the roles of kisspeptin as a key integrator of sexually relevant olfactory stimuli with limbic pathways and the HPG axis in several species including humans (Summarised in **Figure 1**).

### Audition

Beyond olfaction, acoustic signals are crucial for sexual communication, mate selection and ultimately coordinating reproduction (50). Curiously, abundant kisspeptin receptor expression is present in the rodent dorsal cochlear nucleus (33), which may reveal a plausible role for kisspeptin-signalling in auditory processing.

#### Preclinical Animal Studies

In rodents, the presence of a receptive female results in the male emitting song-like ultrasonic vocalisations (USV) to convey information about his motivational state and enhance female sexual approach (51). In terms of reproductive outcomes, a positive relationship between the number of female deliveries and the number of USV syllables emitted by male mice has been observed, revealing that breeding pairs in which males can emit USVs more frequently have more offspring (52). In keeping with this, female mice show more approach behaviour towards vocalising males than devocalised males (achieved by inferior laryngeal nerve sectioning). In a further experiment, female mice were placed in a soundproof chamber in a soundproof room and exposed to an audio file repeatedly for 20 minutes which consisted of either male mice USVs or control noise. Using this experimental paradigm, ARC kisspeptin neuronal activity (determined using dual-label immunocytochemistry with pCREB) significantly increased following exposure to male USVs, whereas neuronal activity in the AVPV was unaffected. Moreover, a moderate positive correlation was identified between ARC kisspeptin neuronal activity and the duration of female searching behaviour during the first five minutes of the USV playback, suggesting that the female’s approaching behaviour towards USVs of male mice relates to the activation of kisspeptin neurons (52). Taken together, this highlights that kisspeptin has the property to integrate vocal signals during sexual interaction with the central reproductive hormonal axis in rodents (Summarised in **Figure 1**). Whether vocal signals modulate the downstream release of reproductive hormones remains to be studied.

#### Human Studies

It has been shown that acoustic signals in humans can independently influence the perceived attractiveness of an individual (53). Indeed, clinical studies reveal that women’s preferences for men’s voices peaks around the time of ovulation (54), highlighting the possible involvement of reproductive hormones in the processing of auditory cues of attraction in humans. To date, there have been no human studies assessing the role of kisspeptin in auditory cues of attraction but would be an interesting avenue for future work based on the aforementioned nonhuman data.

### Sexual Partner Preference and Sexual Motivation

In mammalian reproduction, sexually active males seek female conspecifics, while oestrous females approach males, with this sex-specific behaviour termed sexual partner preference (55). An increasing body of work implicates kisspeptin-signalling in the neuroendocrine modulation of sexual partner preference.

#### Preclinical Animal Studies

In a landmark study in 2007 which triggered much of the recent interest in kisspeptin and behaviour, whereas wildtype male mice spend >70% of their investigatory time with female stimulus animals, gonad intact *Kiss1r* knockout (KO) male mice exhibit no preference for either sex and spend an equivalent investigatory time with male and female conspecifics, despite normosmia (56). Of note, testosterone replacement in these mutant mice fails to restore normal partner preference (56), highlighting a key role for the kisspeptin receptor specifically in controlling sexual partner preference. Similarly, using the chemogenetic DREADDs (Designer Receptor Exclusively Activated by Designer Drugs) approach to selectively stimulate kisspeptin neurons in the posterodorsal subnucleus of the MeA (MePD), doubles the time male mice spend investigating an oestrous female over another gonadally-intact male (57).

Moreover, a very recent study examined the effects of kisspeptin on sexual motivation and its dependence on testosterone levels in male rodents (58). In this study, behavioural effects with an oestrous female were evaluated following intranasal administration of a GnRH analogue (buserelin), intraperitoneal kisspeptin or intranasal kisspeptin. Intranasal buserelin increased circulating testosterone levels but did not affect behavioural measures of sexual incentive motivation, whereas intraperitoneal kisspeptin increased both circulating testosterone and sexual motivation. Notably, intranasal kisspeptin also increased sexual motivation, despite not affecting circulating testosterone levels (58). Taken together, these data provide interesting insight for GnRH/testosterone-independent kisspeptin-effects to stimulate sexual motivation in male rodents.

A similar critical role for kisspeptin-signalling in regulating sexual partner preference in female rodents has been observed in a key elegant study. Ovariectomised and hormone-primed *Kiss1* KO mice fail to display normal male-directed preference, whereas a single peripheral injection of kisspeptin resolves this deficit (59). Interestingly, selective viral ablation of AVPV kisspeptin neurons also results in female mice failing to display any male-direct preference, which again resolves following a single peripheral injection of kisspeptin (59), indicating site-specificity of the AVPV for kisspeptins control of mate preference in female mice. Furthermore, exploiting a transgenic GnRH deficient mouse model (which progressively loses GnRH expression during adulthood) results in female mice displaying female rather than male-directed preference, which normalises following a single peripheral injection of GnRH (but not kisspeptin) (59). This indicates that kisspeptin signals through GnRH neurons to specifically mediate sexual partner preference.

#### Human Studies

Visual cues are a fundamental aspect of human sexual attraction, with facial beauty known to provide a symbol of sexual potential and reproductive fitness (60, 61). Moreover, evidence reveals that prefrontal areas, in particular the medial pre-frontal cortex (mPFC), are heavily involved in the human perception of beauty (62–64). Consistent with this, in a recent study of healthy heterosexual men, peripheral kisspeptin administration increased activity in the mPFC and superior frontal gyrus (a further aesthetic brain region), in response to female faces (48). It is interesting that in this study, significant correlations were observed between kisspeptin-enhanced brain activity and relevant psychometric parameters. For instance, men with lower baseline sexual quality of life scores displayed higher brain responses to kisspeptin in the anterior cingulate cortex and insula when viewing less attractive female faces (48). From a functional perspective, these areas are implicated in sexual arousal (65), facial attraction (63, 66) and motivation towards reward (67, 68). Therefore, kisspeptin’s enhancement of these brain regions on viewing less attractive female faces may serve to strengthen feelings of reward, attraction and incentive motivation in individuals experiencing lower sexual quality of life, ultimately to encourage reproduction at a population level.

### Copulatory Behaviour and Arousal

In female mammals, sexual receptivity (as a proxy for sexual motivation) relates to behavioural strategies required for fertile copulation with a male. It comprises the adoption of a posture which facilitates intravaginal ejaculation to occur, termed lordosis (69). In a sexually receptive female rodent, lordosis is characterised by dorsal flexion of the back and an immobile posture in response to stimulation by male mounting (70). Similarly, male sexual and copulatory behaviours include a succession of behavioural responses, which in rodents includes mounting, thrusting and intromitting behaviours before reaching ejaculation (71).

#### Preclinical Animal Studies

The MeA is a key brain region involved in sexual behaviour. Lesioning of the MeA in rats suppresses male copulatory behaviours such as erections and intromissions (72, 73). Although androgen receptors are present in the MeA (74), direct administration of androgens does not cause spontaneous erections (75), suggesting that additional factors are required to stimulate this physiological pathway. In keeping with this, microinjections of kisspeptin directly into the MeA of male rats dose-dependently stimulates ex-copula erections, an effect blocked by pre-treatment with a kisspeptin receptor antagonist (76). Furthermore, when kisspeptin is infused into the intracerebroventricular space, no erections are observed (despite comparable rises in circulating LH), demonstrating site-specificity of the MeA for kisspeptin’s effects on rodent erections and crucially suggests its role in this aspect of sexual behaviour is independent of downstream GnRH, LH or testosterone (76).

Regarding female rodents, in *Kiss1* KO female mice, there is significant impairment of lordosis behaviour, which is rescued by a single peripheral injection of kisspeptin (59). Peripheral and direct intracerebroventricular injection of kisspeptin are also able to induce the lordosis reflex in wildtype adult females, highlighting the ability of peripheral kisspeptin to cross into the brain (59). A population of kisspeptin neurons in the rostral periventricular area of the third ventricle (RP3V) appear to have a central role in this behaviour as selective ablation of RP3V neurons leads to profound deficits in lordosis, whereas optogenetic stimulation of these neurons activates lordosis behaviour (59). A particularly important observation in this study was that kisspeptin’s effects on lordosis are GnRH-independent as GnRH is not able to induce lordosis behaviour in *Kiss1* knockout females (59). Indeed, tract-tracing reveals that RP3V kisspeptin neurons project to another population of kisspeptin cells in the ventrolateral part of the ventromedial hypothalamus, which express neuronal nitric oxide synthase (nNOS) (59). Peripheral kisspeptin administration does not stimulate lordosis in nNOS knockout females, however, co-administration of kisspeptin with a nitric oxide donor is able to restore lordosis behaviour (59). More recently, the same group showed that direct injection of kisspeptin into the ventromedial hypothalamus stimulates lordosis, whereas direct injection of an nNOS inhibitor decreases lordosis (77). By contrast, no effect on lordosis is observed when kisspeptin is injected into the paraventricular nucleus (77). Taken together, this suggests that kisspeptin action *via* nitric oxide signalling in the ventromedial hypothalamus is essential for sexual behaviour in female mice.

#### Human Studies

In healthy heterosexual men, peripheral administration of kisspeptin enhances limbic brain activity (as determined by fMRI) in response to visual sexual stimuli, including the anterior and posterior cingulate and amygdala (78). Given that desire for sexual stimulation is an important component of the human sexual response (79), it is intriguing that in this study the greater kisspeptin enhanced limbic brain activity (including in the putamen) to sexual images, the less aversion to sex healthy men displayed (determined using behavioural tests). Moreover, in response to sexual images, kisspeptin was observed to activate limbic structures (including the hippocampus) more in men with lower baseline reward behavioural scores, which is highly relevant given that human sexual behaviour is closely associated with pleasure and reward. Of note, very recent data reveals that using the same administration protocol, kisspeptin does not affect brain responses to visual food stimuli or appetite parameters in healthy young men, assessed by fMRI and psychometric tests respectively (80), indicating that kisspeptin’s effects on limbic brain regions are specific to sexual and emotional stimuli. Furthermore, this latter recent study suggests that the effects of kisspeptin on human metabolism may be predominantly driven by changes in energy expenditure (81).

In addition to modulating human brain processing during stimulatory tasks, kisspeptin’s effects on resting brain connectivity have also been explored. Resting brain connectivity constitutes an important element of human behaviour (82) with the default mode network being the principle of these (83). In a subsequent fMRI analysis of healthy heterosexual men, peripheral administration of kisspeptin was observed to modulate the default mode network, which correlated with enhanced limbic brain activity in response to visual sexual images (84). Moreover, kisspeptin’s modulation of this network was greater in men with less reward drive and correlated with reduced sexual aversion (84). To date, there have been no human studies of kisspeptin’s effects on female sexual behaviour.

Collectively, experimental evidence reveals an important neuromodulatory role for kisspeptin-signalling in copulatory and sexual behaviours in rodents. In addition, clinical studies illustrate the emerging influence of kisspeptin in human sexual and emotional brain processing. Given these interesting findings, studies in patients with psychosexual dysfunction to translate these findings for patient benefit, are eagerly awaited (Summarised in **Figure 1**).

### Bonding Behaviour

Attachment and emotional union with a partner are important components in mammalian reproduction (85). To date, kisspeptins role in bonding behaviour has only been explored in humans. Using fMRI, peripheral administration of kisspeptin to healthy men has been shown to enhance limbic brain activity, in response to non-sexual couple-bonding images (78). Specifically, this was observed to occur in the thalamus and globus pallidus, which are brain regions implicated in romantic love (86, 87), as well as the amygdala, also implicated in bonding (87). From a functional perspective, kisspeptin’s enhanced activation of the amygdala correlated with improvements in positive mood (78), implicating kisspeptin in the processing of bonding stimuli, which is important in driving reproduction from a behavioural level.

## Mood and Emotions

Mood and emotions are important prerequisites for optimal reproductive health. In fact, mood disorders in humans are frequently associated with low sexual desire (88) and decreased fertility rates (89). In view of the intricate crosstalk between reproduction, mood and emotions, it is plausible that they are regulated by similar hormonal mechanisms.

### Depression

#### Preclinical Animal Studies

Kisspeptins antidepressant-like effects have been identified in male mice using a modified forced swimming paradigm (90). During this test, rodents are placed into a container filled with water and assessed for active (swimming and climbing) and passive (immobility) behaviours, suggestive of anti-depressant and depressant effects, respectively (91). This study revealed that intracerebroventricular delivery of kisspeptin increased antidepressant-like behaviours (climbing and swimming times), whilst reducing the duration of immobility (90). Regarding interplay with downstream systems, pre-treatment with a nonselective α-adrenergic, α2-adrenergic or nonselective 5-HT2 serotonergic receptor blocker abolished kisspeptins anti-depressive actions, whereas pre-treatment with a cholinergic, dopaminergic or GABAergic receptor blocker had no effect on kisspeptins actions. Therefore, the results from these receptor blockade experiments indicate that kisspeptin can interplay with downstream adrenergic and serotonergic systems to bring about antidepressant-like effects (90).

Given the interaction between kisspeptin and the serotonergic system, it is interesting to consider the brain regions involved in eliciting the antidepressant-like effects. In a recent study, adult male rats received four weeks of intraperitoneal escitalopram (92), a widely prescribed selective serotonin reuptake inhibitor for mood disorders, which increases serotonin activity in the brain by limiting its reabsorption. This experimental paradigm significantly upregulated *Kiss1* mRNA expression in the amygdala (272% increase compared to saline treated rats). Furthermore, *Kiss1r* mRNA was highly increased in the hypothalamus, hippocampus and cerebellum by 170%, 177% and 131%, respectively (92). Indeed, these findings are congruent with a previous study demonstrating that intra-cerebroventricular injections of serotonin hydrochloride to male rats significantly enhances hypothalamic kisspeptin expression and resultant circulating LH concentrations (93). Collectively, these data garner further evidence for the interplay between kisspeptin and the serotonergic system in eliciting antidepressant actions, as well as evidence for the putative brain regions involved.

#### Human Studies

In healthy young men peripheral administration of kisspeptin does not appear to affect positive mood during psychometric testing, whereas administration does reduce negative mood (78), in keeping with the aforementioned antidepressant-like effects in rodents (90). From a mechanistic perspective, brain responses (determined using fMRI) in this study revealed that kisspeptin administration enhanced frontal brain activity in response to participants specifically viewing negative images (e.g. pictures of car crash scenes) (78). This is especially pertinent given that frontal brain activity is important in human negative-mood regulation (94). Taken together, these studies in rodents and humans reveal antidepressant-like effects from kisspeptin-signalling (Summarised in **Figure 1**). An exciting direction for future clinical investigation will be determining whether kisspeptin’s positive stimulatory effects can be recapitulated in patients with mood disorders.

### Anxiety

#### Preclinical Animal Studies

Similar to mood, anxiety and reproductive health are closely related, with anxiety an established trigger for sexual dysfunction (95). However, unlike the experimental evidence revealing an unambiguous and positive modulatory effect for kisspeptin-signalling in depression, its influence on anxiety-like behaviour remains to be fully clarified.

Intracranial administration of kisspeptin to zebrafish increases the number of top-to-bottom transitions during a novel tank diving test, indicating stimulatory exploratory behaviour and attenuated anxiety (96). Moreover, this was associated with a significant increase in *c-fos* mRNA expression in the ventral habenula (96), an evolutionarily conserved structure in the brain of vertebrates implicated in emotional decision-making (16). Regarding rodents, selectively stimulating MePD kisspeptin neurons in male mice using the pharmacosynthetic DREADDs approach has been observed to enhance social interaction between the test mouse and a same sex juvenile conspecific during a 5-minute study period by >50% (57). Using this experimental approach, selectively stimulating kisspeptin neurons in MePD also increases exploratory time in the open arms of an elevated plus maze (EPM) test almost 15-fold, indicating an anxiolytic effect (57).

Comparatively, other studies suggest anxiety-like behaviour related to kisspeptin. In male rats, central administration of kisspeptin dose-dependently reduces the number of entries into and the time spent in the open arms an EPM test, whilst inducing hyperthermia, spontaneous locomotor activity and dose-dependently increasing basal corticosterone levels, suggesting anxiogenic-like kisspeptin effects (97). Furthermore, anxiety-associated behaviour has been examined by generating a *Kiss1r*-deleted murine model, whereby the kisspeptin receptor was globally deleted, but rescued selectively in GnRH neurons, to ensure normal testosterone levels (98). This resulted in male mice spending twice as long in the open arms of an EPM (without affecting performance in the open field test), which may indicate that intact kisspeptin-signalling enhances anxiogenic responses to heights (98). Moreover, a recent study investigated whether central administration of the octapeptide fragment kisspeptin-8 influences anxiety responses in rodents (99). In this study, adult male rats displayed anxiogenic effects as suggested by decreased time in the open arm of an EPM and increased corticosterone levels (99). While kisspeptin can bind and activate neuropeptide FF receptors (100), which have been implicated in anxiety (101), administration of kisspeptin-8 in this study increased LH levels, suggesting kisspeptin receptor activation (99).

In contrast, further experiments suggest that kisspeptin has no modulatory effects on anxiety responses in rodents. In male rats, intraperitoneal administration of kisspeptin does not affect levels of plasma adrenocorticotropic hormone and corticosterone under basal or stress-induced conditions (achieved by 30-minutes of restraint in a plastic rodent restraint tube) (102). Similarly, central administration of kisspeptin to male rats has been observed not to influence stress-related behaviours, including locomotive, grooming and sleeping behaviour (103).

#### Human Studies

Congruent with the latter rodent data, peripheral administration of kisspeptin in two separate clinical studies has been observed not to affect psychometric measures of anxiety or circulating cortisol levels in healthy men (48, 78).

Taken together, this indicates that depending on the species examined, the route of kisspeptin administration and the experimental model employed, kisspeptin-signalling may have an anxiolytic (57, 96), anxiogenic (97–99) or have no effects on anxiety (48, 78, 102, 103) (Summarised in **Figure 1**). Further studies (particularly in rodents where results are conflicting) are therefore required to clarify this contradictory area in kisspeptin biology. However, human data is currently reassuring given the future potential for kisspeptin-based therapeutics.

### Fear

Fear is associated with impaired reproductive performance (104), triggering studies examining the influence of kisspeptin-signalling in modulating fear.

#### Preclinical Animal Studies

In zebrafish, both *Kiss1* and *Kiss1r* are co-expressed in the ventral habenula (17, 18). From this key evolutionary conserved brain structure, Kiss1 and Kiss1r neuronal projections are sent to the median raphe (105), which resides near serotonergic neurons (106). By means of an alarm-substance-evoked fear paradigm in zebrafish, intracranial administration of Kiss1 (Kiss1-15), but not Kiss2 (Kiss2-10), has been observed to reduce erratic movements and freezing behaviour (i.e, behavioural parameters indicative of fear) and dose-dependently increase serotonin-related gene expression (*pet-1* and *slc6a4a* mRNA levels), demonstrating that habenula kisspeptin modulates the serotonergic system to attenuate fear (96). Moreover, pharmacological blockade using methysergide (a 5-HT1A and 5-HT2 receptor antagonist) blocks the effect of intracranial kisspeptin administration to reduce fear (105). Taken together, these data highlight that interplay between kisspeptin-signalling and the serotonin system (via 5-HT1A and 5-HT2 receptors) decreases the fear response in zebrafish (Summarised in **Figure 1**). In view of these findings, further studies are warranted to examine the fear-altering effects of kisspeptin in non-piscine species, such as humans where no studies have currently been performed as yet.

## Interplay with Downstream Systems to Modulate Behaviour

Given the central role of kisspeptin-signalling in governing the aforementioned essential behaviours required for successful reproduction, it is interesting to consider the possible mechanisms. Kisspeptin may be directly acting on kisspeptin receptors expressed in key limbic and paralimbic brain structures (8–11) to regulate behaviour or indirectly through downstream reproductive signals, although as discussed above there is robust experimental evidence of roles independent of these downstream reproductive signals (such as GnRH, LH and gonadal steroids). In addition, kisspeptin also interacts with a plethora of additional downstream neuroendocrine systems as discussed below.

### Preclinical Animal Studies

In the rodent amygdala, fluorescence immunochemistry reveals that kisspeptin neurons reside in close apposition and receive putative innervation from vasopressinergic and dopaminergic neurons (41). Furthermore, MeA kisspeptin neurons are a mixed population in which 71% express markers for GABAergic (Vgat) and 29% for glutamatergic (Vglut2) neurotransmission in male mice (44). In view of the established functions these neurotransmitter systems have in regulating behaviour (107–109), could suggest their interplay with kisspeptin-signalling to bring about these behavioural effects.

Regarding female rodents, whereas sexual partner preference depends on downstream GnRH-signalling (59), interplay with NO in the ventromedial hypothalamus is vital for kisspeptins role in regulating the key reproductive behaviour of lordosis (77). Moreover, kisspeptin induces antidepressant-like actions in rodents through adrenergic and serotonergic systems (90). Consistent with this, kisspeptin neurons in the habenula modulates the serotonergic system to suppress the fear response in zebrafish (96, 105).

### Human Studies

Until recently, there was no data regarding kisspeptin’s interplay with downstream neuropeptide or neurotransmitters outside of the reproductive hormonal axis in humans. Of note, a range of animal models reveal that kisspeptin interacts with the key inhibitory neurotransmitter gamma-aminobutyric acid (GABA) (110–114). In keeping with this, a recent study employed proton magnetic resonance spectroscopy to examine the *in vivo* effects of kisspeptin administration on central GABA levels in the human brain (115). Using this approach, peripheral kisspeptin administration resulted in a 15% decrease in total endogenous GABA concentrations in the anterior cingulate cortex of healthy men (115). This is particularly relevant given that a comparable magnitude of GABA change has previously been reported in psychological studies with functional significance (116, 117).

Collectively, these data reveal that while the mood and behavioural effects of kisspeptin may be the direct action of kisspeptin on its receptor, they may also be the result of interplay between kisspeptin and these additional downstream neuropeptides and neurotransmitter systems with kisspeptin serving as the overall conductor. Further studies examining other downstream neuroendocrine systems of interest are warranted in humans, particularly given the recent *in vivo* data provided by proton magnetic resonance spectroscopy.

## Conclusion

Reproduction and the related behavioural strategies that are required to produce offspring are intricately linked processes. Indeed, disturbances in these behaviours can have detrimental effects on reproduction, suggesting coordination through similar hormonal mechanisms. Furthering our knowledge in this regard has major clinical significance, given the prevalence of psychosexual dysfunction (118) and the associated detrimental effects on quality of life, interpersonal relationships and fertility (88, 119, 120).

The reproductive neuropeptide kisspeptin sits at the apex of the HPG axis, where it has important roles in controlling downstream reproductive hormone secretion (6, 7). Beyond the hypothalamus, kisspeptin and its receptor are located throughout the rodent and human limbic systems (8–11), providing an anatomical framework for the important modulatory roles kisspeptin-signalling plays in reproductive behaviour. As discussed throughout this review, data from animal models reveals stimulatory effects on all aspects from olfactory-mediated sexual partner preference and copulatory behaviours, through to controlling mood and emotions in a presumed drive towards reproduction. Crucially, current limitations in the studies of kisspeptin-signalling in behavioural modulation need to be acknowledged. For instance, kisspeptin can potentially modulate activity in several brain regions based on its gene and receptor expression pattern as well as human functional MRI findings above. However, the importance of distinct kisspeptin populations beyond the studied hypothalamus, amygdala and hippocampus (such as the thalamus) with regards to behavioural roles remain to be clarified in animal models. Indeed, the application of novel chemogenetic (such as DREADDs) or optogenetic techniques to selectively stimulate or inhibit kisspeptin neuronal populations (44, 57) will allow investigation of endogenous kisspeptin-signalling in these unexplored brain regions in future. This is scientifically important as although human functional MRI studies have identified several brain regions involved in behaviour following kisspeptin administration, the precise neuronal subtypes (e.g. kisspeptin, GABA, dopamine etc) cannot be identified by fMRI studies. However, recent proton magnetic spectroscopy studies (for GABA) have provided some recent human data in this regard (115). An additional limitation of the behavioural studies so far is that certain behaviours have been investigated exclusively in one sex (e.g., anxiety in male animals/humans, or human sexual behaviour as yet only in men), therefore additional studies are warranted to examine for sexual dimorphisms.

Building on the animal literature and from a translational perspective, modern functional neuroimaging techniques (such as fMRI and spectroscopy) have been indispensable to investigate the role of kisspeptin in human sexual and emotional brain processing. Given these findings, studies examining kisspeptin in patients with psychosexual and mood disorders are much needed to improve our knowledge of the neuromodulatory effects of kisspeptin in health and disease. To this end, combined with the available updated literature, these data would provide fundamental mechanistic and pharmacological insight for the neurophysiological actions of kisspeptin and unlock kisspeptin as a therapeutic target for the management of reproductive, and related psychological and psychosexual disorders.

## Author Contributions

EM and LY, researched the material and wrote the first draft. EM and LY, joint first authors. WD and AC, co-corresponding and senior authors. All authors contributed to the article and approved the submitted version.

## Funding

This article presents independent research funded by the Medical Research Council (MRC) and supported by National Institute for Health Research (NIHR) Imperial Biomedical Research Centre and NIHR Clinical Research Facility. The views expressed are those of the authors and not necessarily those of the MRC, NIHR or the Department of Health.

EM and LY are funded by MRC Clinical Research Training Fellowships (MR/T006242/1 and MR/R000484/1), AA by an NIHR Clinician Scientist Fellowship (CS-2018-18-ST2–002), WD by an NIHR Research Professorship (NIHR RP-2014-05-001) and NIHR Senior Investigator Award, and AC by the National Health Service.

## Conflict of Interest

The authors declare that the research was conducted in the absence of any commercial or financial relationships that could be construed as a potential conflict of interest.

## Publisher’s Note

All claims expressed in this article are solely those of the authors and do not necessarily represent those of their affiliated organizations, or those of the publisher, the editors and the reviewers. Any product that may be evaluated in this article, or claim that may be made by its manufacturer, is not guaranteed or endorsed by the publisher.
